# Mechano-catalytic conversion of polypropylene over zeolite-based materials

**DOI:** 10.1039/d5cy00935a

**Published:** 2025-10-24

**Authors:** Adrian H. Hergesell, Claire L. Seitzinger, Hubert Pasternak, Laura Seidling, Viviana M. Ospina Guarin, Nicole Karpensky, Florian Puch, Thomas Welzel, Ina Vollmer

**Affiliations:** a Inorganic Chemistry and Catalysis Group, Institute for Sustainable and Circular Chemistry, Utrecht University The Netherlands i.vollmer@uu.nl; b carboliq GmbH Remscheid Germany; c Thüringisches Institut für Textil- und Kunststoff-Forschung e.V. Rudolstadt Germany; d Plastics Technology Group, Faculty of Mechanical Engineering, Technische Universität Ilmenau Germany

## Abstract

Chemical recycling can convert polymers into useful chemicals. Polyolefins can be chemically recycled into their monomers and other hydrocarbons *via* catalytic pyrolysis or mechano-chemistry. While pyrolysis catalysts are highly active but not selective, mechano-chemistry is more selective but lacks quantitative yields. To address these issues and unlock potential synergies, we herein investigate the effect of zeolite-based pyrolysis catalysts on the conversion of polypropylene during ball milling at room temperature and elevated temperatures, as well as during catalytic kneading. Initially, zeolite catalysts are highly active in the ball mill and their activity is dependent on acid site density. However, they deactivate quickly under the harsh collisions in the ball mill due to the collapse of their crystalline framework. To circumvent deactivation, we used the concept of direct mechano-catalysis and immobilized the zeolite material on surface-roughened grinding spheres. This effectively protects active sites against contact with the container wall or other grinding spheres while allowing contact with polypropylene, leading to sustained catalytic activity and requiring much lower amounts of zeolite. In addition, catalytic kneading of molten polypropylene was investigated as an alternative where energy input is more uniformly distributed in the volume and time compared to highly localized and forceful impacts within the ball mill. Although a synergy of thermo- and mechano-chemical effects was observed initially, the energy intake was limited by a fast decline of melt viscosity due to polymer backbone cleavage. Mechano-chemical conversion and catalytic pyrolysis of polyolefins are two promising platforms for chemical recycling. Our study illustrates the difficulties in combining both and possible pathways to overcome these challenges.

## Abstract 2

Ball milling or kneading of polypropylene with zeolite enforces contact during mechano-chemical recycling. Zeolite instability and low impact efficiency can be addressed by attaching zeolite to spheres to create surface-activated mechano-catalysts.

## Graphical abstract

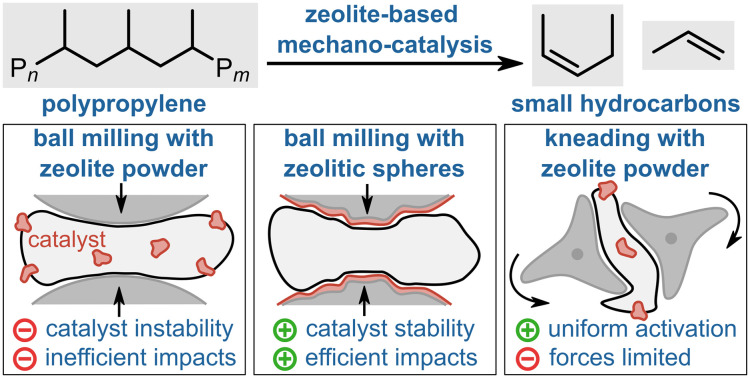

## 1. Introduction

Chemical recycling of plastic waste to small molecules, such as monomers, can be used to make new plastics with virgin quality.^1^ For the most produced plastics, *i.e.*, polyolefins such as polyethylene (PE) and polypropylene (PP), current chemical recycling is based on unselective high-temperature pyrolysis.^1^ This process, however, leads to low-value hydrocarbon mixtures which cannot be used without further upgrading.^1,2^ Solid acid materials, such as zeolites, have been used as catalysts and can increase the selectivity towards, *e.g.*, valuable aromatics.^2,3^ The majority of these catalysts, however, were originally designed and optimized for the gas-phase conversion of short hydrocarbons, which behave differently than high molecular weight polymers.^4,5^ In contrast to short hydrocarbons, for example, polyolefins are not able to fully infiltrate the nanometer-sized pore systems of typical catalysts such as commonly employed zeolites. These transport limitations are caused by the small catalyst pore diameters compared to the large size of macromolecules which also makes the plastics highly cohesive and viscous.^4,6–11^ Ultimately, this causes lower catalyst utilization, energy efficiency, and overall productivity.^4,6^ In addition, transport limitations are especially relevant for more realistic higher molecular weight polyolefins.^4,6^

To relieve transport limitations in catalytic processes, mechano-catalysis as the combination of mechano-chemical conditions and the addition of a catalyst is a powerful tool to enforce contact between reactants.^12,13^ Mechano-chemical conversion can be performed in different reactor geometries, such as ball mills, extruders, resonant acoustic mixers, and kneaders.^14–17^ Ball mills rely on loose grinding spheres in a vigorously shaken vessel. These usually hard and dense spheres locally impact the milled material by transferring their kinetic energy.^18,19^ In contrast, reactive extrusion^16,20^ and polymer kneading typically rely on the application of shear forces. These are less locally focused than impacts in the ball mill and readily dissipate through the viscous plastic material. This key difference between extruders and ball mills can lead to lower overall energy inputs and lower mechano-chemical reactivity.^21^ However, a unified framework for the direct comparison of different mechano-chemical techniques is still lacking and motivates further investigations into mechano-chemical activation principles and precise modeling of force distributions.

Mechano-catalytic ball milling as a tool to force contacts between substrates and catalysts has been used for the solvent-free conversion of solid biomass feedstocks.^13,22,23^ With respect to polymer degradation, mechano-catalysis was shown to enable the oxidative degradation of PE over an Fe_2_O_3_-based Fenton system,^24^ and the degradation of PE over Al_2_O_3_ in the presence of water.^25^ Furthermore, our group recently reported on the conversion of PP and PE using surface-activated mechano-catalysts (SAM catalysts).^26^ These catalytically functionalized grinding spheres were obtained by treatment in sulfuric acid and tungstation of zirconia grinding spheres and increased depolymerization rates significantly. In addition to potentially enabling better catalyst–polymer contact *via* mechanical force, ball milling decreases the molecular weight of polymers due to backbone cleavage.^26,27^ This effect could pre-crack polymer chains mechano-chemically and increase their mobility enough to enable additional contact with the catalyst, thereby increasing catalytic efficiency. A conceptually similar effect, where large molecules are first pre-cracked to subsequently reach narrower catalyst pores, contributes to the activity of, *e.g.*, fluid catalytic cracking (FCC) catalysts.^2^

Activity in the catalytic cracking of polyolefins is connected to acidity.^5,28,29^ Most importantly, Brønsted acid sites transform polymer materials into carbocation intermediates which can undergo scission, rearrangement and cyclization reactions to yield smaller hydrocarbons.^30^ In contrast, mechano-chemical degradation of polyolefins occurs *via* radical intermediates.^26,31–33^ These are formed by homolytic scission of polymer backbone bonds under stress and can then undergo follow-up reactions such as depolymerization, transfer, or termination, leading to monomers and other hydrocarbons.^19,25,26,31^ The dominant mechanisms and intermediates of thermo-catalytic and mechano-chemical polyolefin conversion are, therefore, fundamentally different. With respect to a potential interplay of radicals and cations over zeolite-based catalysts, however, radical cations, *i.e.*, species having both radical and cation characteristics at the same time, have been discussed.^34,35^ For example, the formation of such radical cations can be observed *via* electron paramagnetic resonance spectroscopy when reacting alkenes^36^ and alkanes^37^ with zeolites.^34^ Furthermore, the strong electrostatic fields within zeolites combined with their rigidity make them good matrices for the stabilization of such reactive species.^34^ With respect to the reactivity of radical cations, complex sequences of rearrangement, fragmentation, coupling, oxidation, and deprotonation are frequently observed.^34^ Besides zeolites, the reactivity of (acidic) sulfated zirconia catalysts has also been discussed in terms of radical cations.^38,39^

Given that both zeolite-based catalytic conversion and mechano-chemical degradation are promising technologies for the chemical recycling of polyolefins, their combination could offer synergistic benefits. More specifically, the enhancement of catalyst–polymer interactions, mechano-chemical pre-cracking, or combined mechanisms, such as *via* radical cations, could improve yields and/or selectivities. Therefore, in this study, we investigate the influence of zeolite-based catalysts on the mechano-chemical conversion of PP to short hydrocarbons. We performed mechano-catalytic experiments in a ball mill and a polymer kneader and analyzed the activity of catalysts by analyzing products *via* online gas chromatography (GC) and mass spectrometry (MS).

## 2. Experimental

### 2.1 Materials

The zeolite Y materials CBV 400 (SiO_2_ : Al_2_O_3_ = 5.1, denoted as Y-05), CBV 720 (SiO_2_ : Al_2_O_3_ = 30, denoted as Y-30), and CBV 760 (SiO_2_ : Al_2_O_3_ = 60, denoted as Y-60) were acquired from Zeolyst International and used as received. Thorough characterization of these materials was performed previously.^5^

In addition, an equilibrium catalyst (ECAT) from the FCC process was used. This catalyst has been thoroughly characterized previously.^2^ The pristine FCC catalyst consists of a microporous zeolite Y material and silica–alumina domains, held together by clay as a binder. During FCC operation, Fe, Ni, and V species are deposited on the catalyst, and the zeolitic domains are deactivated due to steaming in the regenerator unit and coking. Prior to its use in mechano-catalytic experiments, the ECAT material was calcined in static air to remove organic residues *via* the following procedure: 5 °C min^−1^ to 120 °C, then 20 min at 120 °C, then 10 °C min^−1^ to 550 °C, and then 5 h at 550 °C.

For all ball milling experiments, PP supplied by Ducor Petrochemicals was used, while PP supplied by Sigma-Aldrich was used for high-temperature kneading experiments (see Table 1 for further information).

**Table 1:** PP materials used for mechano-catalytic experiments

Used for	Source	Product	Shape	*M*_n_ (g mol^−1^)	*M*_w_ (g mol^−1^)
Ball milling	Ducor	DuPure G72TF	Powder	83 600^a^	456 900^a^
Kneading	Sigma-Aldrich	427 888	Pellets	37 000	246 000

a Literature^26^ value.

### 2.2 Synthesis of catalytic grinding spheres

To immobilize zeolite on zirconia grinding spheres, sol–gel dip coating with tetraethyl orthosilicate (TEOS) as a binder was used.^40,41^ Commercial 10 mm ZrO_2_ spheres (Zhonglong Materials) were first sandblasted with brown fused alumina particles (FEPA F40, *ca.* 400 μm) for 1 min at 6 bar air pressure using a ZionAir SBC990 sand blaster to create surface roughness. Subsequently, spheres were washed with distilled water and dried overnight in air. A solution of TEOS (Sigma Aldrich, 99.0%), distilled water, and ethanol (VWR chemicals) was prepared with a volumetric ratio of 0.5 : 0.5 : 9, acidified with nitric acid (VWR chemicals, 65%) to pH ≈ 2, and mixed well. The spheres were dipped into the solution for 5 min, removed, and dried for 10 min in air. Subsequently, the sequence of dipping, removing, and drying was repeated once. Afterwards, the spheres were dipped again, removed, and then directly covered in CBV 400 powder (Y-05, SiO_2_ : Al_2_O_3_ = 5.1, crystal size: *ca.* 1 μm,^5^ external surface area: 76 m^2^ g^−1^). Finally, calcination was performed at 500 °C for 3 h in static air (heating rate: 2.5 °C min^−1^). Excess CBV 400 was removed prior to use in ball milling experiments.

### 2.3 Ball milling experiments

All ball milling experiments were performed at 30 Hz using a Retsch MM500 vario mixer mill with a 25 ml tungsten carbide (Retsch, 94% WC and 6% Co) container. To allow for a gas flow (12.5 ml min^−1^ N_2_) through the system to remove products, holes were drilled into the commercial container *via* electrical discharge machining, and 1/8′′ Swagelok connections were welded to it. In a typical experiment, PP was loaded together with commercial 10 mm stainless steel grinding spheres (denoted as Fe, Retsch, X46Cr13 (1.4034), 12.5–14.5% Cr, 0.42–0.5% C) or zirconia grinding spheres (Zhonglong Materials) and catalyst powder. The ball-to-powder mass ratio was 19.0 for low-temperature experiments with 6 ZrO_2_ spheres, 24.6 for low-temperature experiments with 6 Fe spheres, and 9.8 for high-temperature experiments with 5 Fe spheres.

The amounts of PP and catalyst are usually indicated as wt% (see Table 2 for absolute amounts). A Teflon sealing ring was used and the container was tightly closed using a wrench.

**Table 2:** Catalyst weight loadings used and corresponding absolute amounts of PP and catalyst

Loading (wt%)	Mass of PP (g)	Mass of catalyst (g)
0	1.00	0.00
7	1.00	0.08
20	0.80	0.20
33	0.66	0.33
50	0.50	0.50

To control the temperature during high-temperature ball milling experiments, the container was wrapped in a flexible glass yarn-insulated heating cable (Horst, 1.0 m, 100 W), attaching a thermocouple to the container, wrapping both the heating wire and thermocouple in woven fiberglass insulation tape, and fixing all materials with heat-resistant adhesive tape (see Fig. S1 for a photograph). Before starting the milling process, the temperature was ramped up to the desired temperature, *i.e.*, 80, 120, or 160 °C, and the formation of volatile products was recorded during a period of *ca.* 60 min after which milling was started with continued tracking of products.

We used a Global Analyzer Solutions GC equipped with a thermal conductivity detector (TCD) and three flame ionization detectors (FIDs) to analyze gaseous hydrocarbon products eluted from the ball mill with a 12.5 ml min^−1^ N_2_ flow (*F*_N_2__). N_2_ and H_2_ were analyzed on the TCD which was connected to a 2 m × 0.32 mm Rtx-1, 3.0 μm column and a 3 m × 0.32 mm Carboxen1010 column. Hydrocarbons were analyzed on different FIDs coupled to certain columns based on their chain lengths: C_1–3_ were separated on a 3 m × 0.32 mm Rtx-1, 3 μm column and a 15 m × 0.32 mm Al_2_O_3_/Na_2_SO_4_ column; C_4–7_ were separated on a 2 m × 0.28 mm MXT-1, 1 μm column and a 14 m × 0.28 mm MXT-1, 1 μm column; C_5–10_ were separated on a 2 m × 0.28 mm MXT-1, 0.5 μm column and a 15 m × 0.28 mm MXT-1, 0.5 μm column. Changes in total volumetric flow due to the formation of products were corrected by using the constant flow of N_2_ (*F*_N_2__) as an internal standard 
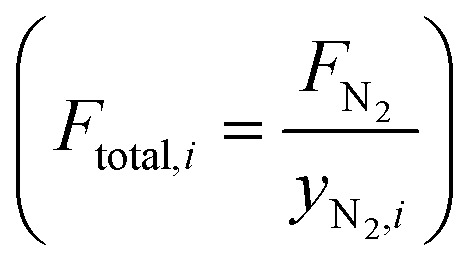
. Molar concentrations of N_2_ during individual injections were calculated by using eqn (1), where *A*_N_2_,*i*_ is the peak area of nitrogen at injection *i*, and using the average of the peak areas of three stable injections before starting the shaking or setting the temperature.1
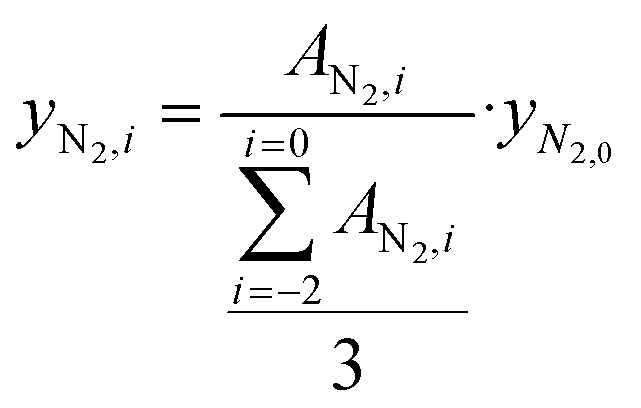
Molar flows of hydrocarbons C_*x*_H_*y*_ with *x* as the carbon number were calculated by using eqn (2).

2 *F*_C_*x*_H__*y*__,*i*_ = *y*_C_*x*_H__*y*__,*i*_·*F*_total_·*x*

Concentrations of individual hydrocarbons were determined according to 
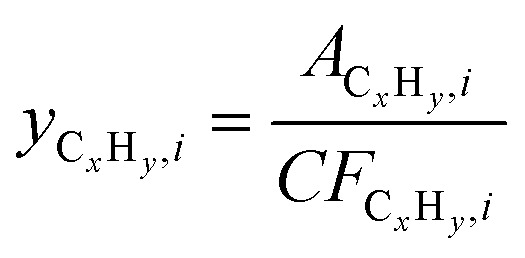
, where *A*_C_*x*_H_*y*_,*i*_ is the hydrocarbon's chromatogram integral and *CF*_C_*x*_H_*y*_,*i*_ is the hydrocarbon's calibration factor. To determine this calibration factor, a GC calibration was performed with a mixture containing known amounts of methane, ethane, propane, butane, heptane and hexane. To obtain the calibration factor, *CF*_C_*x*_H_*y*_,*i*_ = *CF*_C_·*x* was used with *CF*_C_ being the calibration factor normalized by the carbon number, considering that the FID response caused by a hydrocarbon is approximately proportional to its carbon number *x*.

To obtain cumulative hydrocarbon yields, eqn (3) was used which uses the molecular weight of the hydrocarbon *M*_C_*x*_H_*y*__ and integrates the hydrocarbon flow over time.3
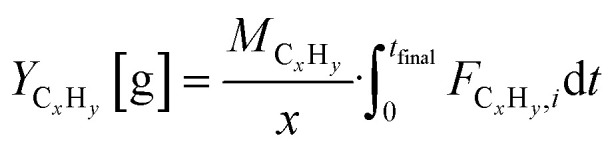


### 2.4 Kneading experiments

Kneading experiments were performed using a Haake Rheomix PolyLab system in the mixer geometry equipped with counterrotating mixing elements (see Fig. S2 for a photograph) and a net volume of the mixing chamber of 69 ml. The temperature was measured using a thermocouple reaching into the plastic melt, and the torque needed for maintaining a set kneading rate (rpm) was continuously recorded. A custom-made chamber cap equipped with a rubber ring was used to close the kneading chamber at the top and to allow for a continuous flow of N_2_ (250 ml min^−1^, residence time: 2 s) *via* a gas inlet and gas outlet.

In a typical experiment, 46 g of PP was kneaded together with or without 2.3 g of ECAT. To this end, the kneading chamber was filled with PP which was molten at 180 °C using a kneading rate of 5 rpm. The chamber was flushed with a flow of 250 ml min^−1^ N_2_ for >10 min. In non-catalytic reactions, the temperature was then directly set to the desired value (220 or 230 °C), and kneading was started at the chosen rate. In catalytic reactions, the slow kneading was stopped, the cap was quickly opened, the ECAT was added, the cap was quickly closed, and the system was flushed again with N_2_, before the temperature was set to the desired value and kneading was started at the chosen rate.

To analyze volatile hydrocarbon products, the N_2_ flow was used to carry them through a stainless steel 1/8′′ connection line heated at 200 °C which ended in an ice-cooled condenser. The gaseous product stream was analyzed with a portable EcoCat (ESS) MS. Masses up to *m*/*z* = 114 were continuously analyzed in multiple ion detection mode, and ion currents of a given mass were divided by the ion current of the inert nitrogen signal (*m*/*z* = 28) to obtain normalized mass spectrometry signals.

### 2.5 Characterization

X-ray diffraction (XRD) was performed on a Bruker 2D PHASER instrument using a LYNXEYE-2 detector and Cu K_α_ radiation at 30 kV and 10 mA. Data were recorded in the 2*θ* range of 5 to 80° with a step size of 0.1°.

Temperature-programmed desorption of ammonia (NH_3_-TPD) was performed on a Micromeritics AutoChem II instrument. To this end, *ca.* 100 mg of sample was first dried under He at 400 °C for 30 min (heating rate: 5 °C min^−1^). Afterwards, NH_3_ adsorption was performed at 100 °C, after which the sample was heated to 700 °C (heating rate: 5 °C min^−1^) to desorb NH_3_.

Scanning electron microscopy (SEM) was performed on a Thermo Scientific Phenom ProX instrument. 10 mm spheres were attached to aluminum holders using double-sided adhesive and conductive carbon tape. An acceleration potential of 10 kV was used to record images.

Nitrogen physisorption experiments were performed on a 3P Instruments Sync 400 at −196 °C after drying at 400 °C for at least 12 h under vacuum. Specific surface areas were determined using the Brunauer–Emmett–Teller method.

Inductively coupled plasma optical emission spectroscopy (ICP-OES) was performed on a Perkin Elmer Avio 500 instrument. Samples were digested with a lithium borate procedure. To this end, a graphite crucible was filled with 100 mg of sample and 600 mg of lithium borate, and heated for 1 h at 975 °C. Subsequently, the mixture was transferred to a beaker containing 250 ml of 2 M nitric acid, and stirred for at least 30 min until full dissolution was achieved. Before analyzing, the solution was passed through a 0.45 μm syringe filter. Measurements were performed with radial plasma using a radio frequency generator at 1500 W.

High-temperature gel permeation chromatography (GPC) was performed on a Polymer Char (Valencia, Spain) GPC-IR instrument using an infrared detector (IR4). Three PLgel Olexis (300 × 7.5 mm, Agilent Technologies) columns were used in series together with a PLgel Olexis (50 × 7.5 mm, Agilent Technologies) guard column. As an eluent, 1,2,4-trichlorobenzene stabilized with butylated hydroxytoluene (300 ppm) was used at a flow rate of 1.0 ml min^−1^. The column temperature was set to 160 °C. PP samples with a concentration of 1 mg ml^−1^ were prepared using heptane as an internal standard. Prior to injection, the samples were dissolved at 160 °C under nitrogen using continuous gentle shaking for 90 min and then filtered. Molar mass distributions were calculated with respect to polystyrene standards (Polymer Char Laboratories, *M*_n_ = 5310 up to *M*_n_ = 1 510 000 g mol^−1^) and were converted to PP equivalents using Mark–Houwink parameters (*α*_PS_ = 0.722, *K*_PS_ = 0.0001016 dl g^−1^, *α*_PP_ = 0.725, *K*_PP_ = 0.0001901 dl g^−1^).

Thermogravimetric analysis (TGA) was performed on a Perkin Elmer TGA 8000 instrument. The ball milling residue was loaded into an alumina crucible and heated to 600 °C using a heating rate of 10 °C min^−1^ and a N_2_ flow of 45 ml min^−1^. The weight loss curves shown are max-normalized.

Offline gas chromatography (GC) on condensed hydrocarbons obtained from kneading experiments was performed on a Varian 430 gas chromatograph equipped with an Agilent CP9013 VF-5 ms (30 m, 0.25 mm, 0.25 μm) column and a 10 m EZ-Guard column, using an injection volume of 0.5 μl. The columns were heated according to the following program: 40 °C to 300 °C at 5 °C min^−1^, and then held at 300 °C for 10 min. To prepare samples, condensed hydrocarbons were removed from the condenser by flushing with 3 ml of dichloromethane (DCM, Sigma-Aldrich, 99.9%). Subsequently, 500 mg of this mixture was combined with 1 ml of DCM and 2 drops of butyl decanoate (Sigma-Aldrich, 98%) as an internal standard.

## 3. Results and discussion

### 3.1 Ball milling of polypropylene and zeolite materials under ambient conditions

We investigated the mechano-catalytic conversion of PP over zeolite-based materials using a shaker mill at 30 Hz.^19^ We modified a commercial 25 ml tungsten carbide container to have a gas inlet and outlet to allow for a continuous flow of 12.5 ml min^−1^ N_2_ for product removal and online analysis. We loaded the container with six 10 mm Fe grinding spheres, PP (*M*_n_ = 84 000 g mol^−1^, *M*_w_ = 457 000 g mol^−1^),^26^ and zeolite powder, and milled for 1 h. While hydrocarbon products up to C_10_ can be detected, we focus our main attention on the lighter products, mainly the monomer propene, due to fast removal from the reactor system.^42^ In contrast, heavier higher hydrocarbons are not removed quantitatively from the reactor at ambient temperatures due to their high boiling point.

**Fig. 1:**
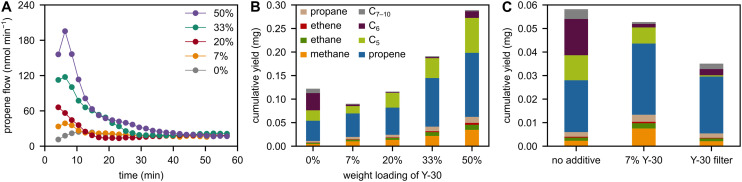
(A) Propene flow during milling of PP with 6 Fe spheres at 30 Hz with 0–50 wt% of Y-30. (B) C_1–10_ hydrocarbon yield obtained after 1 h of milling PP with 6 Fe spheres at 30 Hz with 0–50 wt% of Y-30. (C) C_1–10_ hydrocarbon yield obtained after 0.5 h of milling PP with 6 Fe spheres at 30 Hz under three conditions: (i) without Y-30, (ii) with 7 wt% Y-30, and (iii) without any Y-30 in the reactor, but a Y-30 filter downstream of it.

Milling PP without a catalyst forms hydrocarbons (Fig. 1A and B). Propene as the major product is generated continuously with a rate of *ca.* 20 nmol min^−1^. According to an established mechanism, the formation of products is initiated by backbone scission forming mechano-radicals.^31,32^ From these intermediates, propene is directly generated *via* β scission, while the formation of other hydrocarbons is based on a more complex sequence of scission, radical transfer, and coupling reactions.^19,26^

To investigate the mechano-catalytic effect of zeolites, we added the Brønsted acidic H form of zeolite Y (faujasite framework). The zeolite Y powders used herein are denoted as Y-*n*, with *n* being their molar SiO_2_ : Al_2_O_3_ ratio. Adding Y-30 powder to PP influences the formation rates of propene and other hydrocarbons during milling (Fig. 1A, see Table S1 for replicates). The effect on product formation is strongly dependent on the catalyst loading, with the cumulative hydrocarbon yields after 1 h displaying a maximum when adding 50 wt% of zeolite Y (Fig. 1B). Besides enforcing contact between the polymer and catalyst, we believe that catalytic activity also benefits from freshly exposed and highly reactive surface sites due to breakage of zeolite crystals. The catalytic effect is pronounced especially during initial stages of milling. With a loading of 50 wt% Y-30, the propene formation rate is increased by a factor of 10 compared to the non-catalytic case. However, propene production rapidly decreases while milling and is identical to the non-catalytic level after 40 min, and even earlier for lower loadings of Y-30 (Fig. 1A).

While more propene is formed when adding Y-30, the formation of C_5–10_ products seems to be inhibited in comparison with the non-catalytic experiment (Fig. 1B). With an increase in small hydrocarbon formation and a decline in larger hydrocarbon formation when adding Y-30, it seems possible that the zeolite material cracks heavier hydrocarbon products to lighter ones, especially considering the wide use of zeolites as cracking catalysts.^43^ While we consider that pathway unlikely due to the low temperatures in the ball mill, it could explain the increased propene formation rates. This is especially the case when milling at low loadings of zeolite Y (7 and 20 wt%), where the catalyst does not seem to promote overall productivity but only seems to shift production selectivity from heavier to lighter hydrocarbons (Fig. 1B). Another potential cause for the decrease in apparent formation rates of higher hydrocarbons when adding Y-30 could be the unreactive adsorption of large condensable hydrocarbons on the zeolite material, considering the high surface area of zeolites and their use as sorbents.^44,45^ To investigate this effect, we performed an experiment where we milled without a catalyst, but added a fixed bed of Y-30 after the outlet of the reactor at room temperature (Fig. 1C). In contrast to milling without a catalyst bed, lower concentrations of C_5–10_ hydrocarbons are detected when adding a catalyst downstream of the reactor. However, that decrease does not lead to the formation of additional C_1–3_ species, which would be indicative of cracking reactions to smaller hydrocarbons. Therefore, we conclude that further conversion of C_5–10_ hydrocarbons does not play a significant role in the formation of additional C_1–3_ products. Instead, higher hydrocarbons seem to adsorb on the zeolite material in the ball mill and do not leave the reactor setup at ambient temperatures. The additional C_1–3_ products when milling with catalyst seem to be primary products formed *via* mechano-catalytic activity of the zeolite directly in the ball mill.

**Fig. 2:**
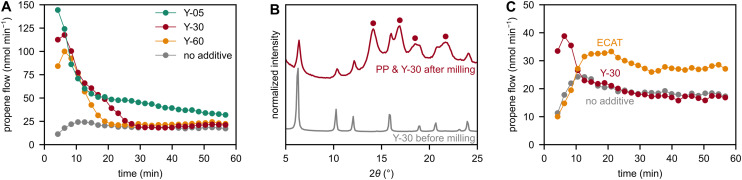
(A) Propene flow during milling of PP with 6 Fe spheres at 30 Hz with 33 wt% of Y-05, Y-30, or Y-60. (B) X-ray diffractograms of fresh Y-30 and a mixture of Y-30 and PP after 1 h of milling PP with 6 Fe spheres at 30 Hz with 50 wt% of Y-30. Reflections caused by PP are marked with dots. (C) Propene flow during milling of PP with 6 Fe spheres at 30 Hz with 7 wt% of Y-30 or 7 wt% of ECAT.

To further understand the reactivity of zeolite materials towards PP during mechano-chemical activation, we varied the acidity of the added zeolites while keeping their loading constant at 33 wt%. The initial formation rates of propene and other hydrocarbons scale with the amount of acid sites present in the zeolite added (Fig. 2A), which is inversely correlated with the molar SiO_2_ : Al_2_O_3_ ratio *n* in Y-*n*. Therefore, the presence of alumina and/or acid sites seems to be an integral factor in the mechano-catalytic formation of hydrocarbons.

With respect to the production profiles of propene (Fig. 1A and 2A), the initial spike and subsequent decline to the non-catalytic level is a motif among all used zeolite Y materials. While the initial high levels are appreciable, the high rates of catalyst deactivation are a point for further investigation. To this end, we performed XRD measurements to understand the structural evolution of zeolites during ball milling. We recorded diffractograms before and after milling of just zeolite, and after milling of zeolite and PP. The diffractogram of fresh Y-30 is characterized by sharp signals which correspond to a highly ordered crystalline material. Milling Y-30 for 1 h with PP, however, leads to broadening of its characteristic reflections (Fig. 2B), which indicates a loss of crystallinity and ordered structure. This is even more evident when milling zeolite without PP for only 10 min (Fig. S3).

It has been reported that ball milling of zeolites can decrease their crystallinity and specific surface area.^46–48^ This behavior also leads to a decrease in acidity, since the strength of Brønsted acid sites is strongly dependent on long-range crystallinity.^47^ In a different study, collapse of the zeolite crystal was shown to both negatively impact the number and strength of acid sites.^47^ In addition, the thermo-catalytic conversion of C_6–8_ aromatics is heavily impeded by pre-milling zeolite Y, with longer milling times causing lower productivities.^48^ Due to consensus in the literature on the loss of acidity and crystallinity during milling, we believe that the loss in structural integrity due to repeated mechanical impact is responsible for the performance loss we observe during ball milling. To probe the loss of acid sites due to ball milling, we performed temperature-programmed desorption of ammonia (NH_3_-TPD) measurements. Ball milling of Y-30 for 10 min at 30 Hz leads to a stark decrease in its acid site concentration from 0.44 mmol g^−1^ to 0.06 mmol g^−1^. In accordance, the specific surface area drops from 788 m^2^ g^−1^ to 14 m^2^ g^−1^ during milling of Y-30. It should be noted that milling pure zeolite subjects the material to much harsher mechano-chemical conditions than milling zeolite with plastic, since the latter provides additional cushioning. Consequently, the observed structural deactivation is expected to be less pronounced during a mechano-catalytic reaction.

The performance loss of zeolites due to mechano-chemical activation is unfortunate, since ball milling relies on exactly these forceful impacts as a chemical driving force, especially for inert polyolefins, which are very difficult to activate mechanically.^19^ While mechano-chemical conversion *via* ambient ball milling and catalytic conversion over zeolites are two methods which are receiving attention for the chemical recycling of polyolefins, their combination, therefore, seems inherently limited by the low stability of zeolites under relevant reaction conditions. In addition, reacting two solids in a ball mill requires the presence of both reactants in a certain volume which is impacted mechanically. However, due to the typically small size of these impacted volumes, the bulk of the material is not sufficiently activated at any given moment. This conceptual drawback can lead to low overall productivity when mechano-chemically converting powder feedstocks using powder catalysts.

**Fig. 3:**
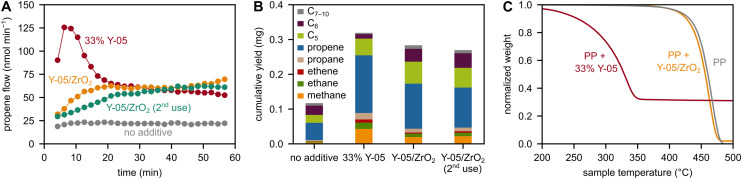
(A) Propene flow during milling of PP with 6 ZrO_2_-based spheres at 30 Hz, using untreated ZrO_2_ without and with 33 wt% of Y-05, and using Y-05/ZrO_2_ grinding spheres. (B) C_1–10_ hydrocarbon yield obtained after 1 h of milling of PP with 6 ZrO_2_-based spheres at 30 Hz, using untreated ZrO_2_ without and with 33 wt% of Y-05, and using Y-05/ZrO_2_ grinding spheres. (C) TGA profiles of untreated PP and of milling residues obtained after 1 h of milling PP at 30 Hz with either 6 untreated ZrO_2_ spheres and 33 wt% of Y-05, or with 6 Y-05/ZrO_2_ grinding spheres.

To address these inherent limitations, we used the concept of direct mechano-catalysis, which describes the catalytic activation of grinding spheres and can enforce catalyst–polymer contacts more efficiently.^12,49^ In addition, the immobilization of catalytically active materials on macroscopic supports can increase mechanical stability, for example when immobilizing metal–organic frameworks on Al_2_O_3_ or SiO_2_ (micro-)spheres.^50,51^ In our case, we developed a new sub-group of surface-activated mechano-catalysts (SAM catalysts) by immobilizing zeolite Y on ceramic zirconia spheres. This was achieved by first sandblasting commercial ZrO_2_ grinding spheres to introduce surface roughness. Subsequently, a commercial zeolite material (Y-05) was attached to the surface by using tetraethyl orthosilicate (TEOS) as a binder, followed by calcination. Milling with Y-05/ZrO_2_ grinding spheres produces more propene and other hydrocarbons than milling with untreated ZrO_2_ spheres, underlining their mechano-catalytic activity (Fig. 3A and B, see Fig. S4 for reference experiments after sandblasting and TEOS treatment). Interestingly, catalytic grinding spheres do not suffer from the typical deactivation pattern of Y-05 and other zeolites and can be reused readily after cleaning with deionized water and drying in air (see Fig. S5 and Table S2 for catalyst recycling experiments). We believe that this increased catalyst stability compared to powder zeolites is connected to the protection of Y-05 sites within the rough surface of grinding spheres. This effect would allow reactive collisions with the plastic material but prohibit the crushing of Y-05 between grinding surfaces.

The catalytic activity of Y-05/ZrO_2_ spheres seems to be directly linked to permanent immobilization of the zeolite material on the surface which localizes catalytic sites directly at the points of mechanical energy input during milling. This is in stark contrast to an alternative hypothesis where Y-05/ZrO_2_ spheres would merely be carriers of the Y-05 material which is released into the plastic mixture upon starting the mill and then acts as a powder catalyst. Indeed, abrasion of surface species and flattening of grinding spheres can be observed *via* scanning electron microscopy (SEM, Fig. S6). In addition, we performed inductively coupled plasma optical emission spectroscopy (ICP-OES) measurements and found that the residue after milling PP for 1 h at 30 Hz with Y-05/ZrO_2_ contains Al and Si in amounts equivalent to 0.02 wt% Al_2_O_3_ and 0.04 wt% SiO_2_, respectively.

However, we believe that the presence of zeolitic species in the powder material is not the main driver for depolymerization since the grinding spheres can be reused with similar activity after separation from the plastic material and cleaning, indicating the retainment of catalytic sites on the surface. In addition, thermogravimetric analysis (TGA) measurements (Fig. 3C) show only 2% of inorganic material in the plastic residue after milling with Y-05/ZrO_2_ (see Fig. S7 for XRD), compared to 31% when milling directly with Y-05 powder, despite similar levels of mechano-catalytic activity. Furthermore, the abrased 2% of inorganic material are thermo-catalytically inactive. Also according to ICP-OES measurements, we instead attribute the majority of this residue to tungsten carbide or the zirconia material from the milling vessel and grinding spheres, respectively, and conclude that the catalytic activity of Y-05/ZrO_2_ is related to immobilized surface sites.

### 3.2 Ball milling of polypropylene and zeolite materials at elevated temperatures

Besides immobilizing the zeolite material on grinding spheres to counter catalyst deactivation, we identified an equilibrium catalyst (ECAT) from the fluid catalytic cracking (FCC) process as a different zeolite-based material which produces more stable hydrocarbon formation rates than zeolite Y during ball milling. More specifically, the mechano-catalytic activity of the ECAT does not abruptly decrease, but is rather stable over 60 min of ball milling (Fig. 2C).

The ECAT is a composite material which contains silica, alumina, clay, and zeolite Y. The latter is present in a heavily deactivated form due to dealumination, framework collapse, and metal poisoning due to FCC operation. Due to the unknown content and the deactivated form of zeolite Y domains in the ECAT, activity comparisons normalized by zeolite content between the ECAT and pure zeolite Y are impractical in our case. In addition, we believe that the mechano-catalytic activity of the ECAT is not governed by its zeolite Y domains due to their deactivated form and the different propene production profiles between the ECAT and pure zeolite Y. Instead, other active sites in the material which might be less prone to collapse or to lose their active site strength upon collapsing are believed to be the active domains of the ECAT. Such sites might include acidic alumina and silica in the catalyst matrix or deposited metals.^2^ In contrast to pure zeolite Y, the ECAT can therefore be utilized as an active mechano-catalyst for extended periods of time. Besides its enhanced stability, the use of the ECAT has the advantage of it being a waste product from the FCC process that would otherwise be discarded.

To unlock potential synergies between mechano- and thermo-catalysis during PP conversion over zeolite-based materials, we performed ball milling experiments at elevated temperatures, *i.e.*, 80–160 °C. We used the same setup as for ball milling at room temperature (RT) but wrapped heating wire and insulation tape around the container and added a thermocouple. We used 2 g of PP together with 5 Fe spheres and 100 mg of Y-30 or ECAT. To isolate the contribution of thermo-catalytic conversion, we started the heating to 80–160 °C *ca.* 1 h before milling while recording the formation of potential products.

**Fig. 4:**
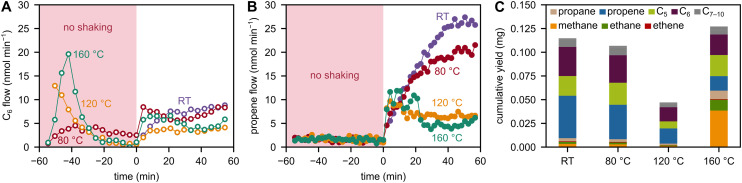
Non-catalytic high-temperature ball milling experiments. (A) C_6_ flow and (B) propene flow during milling of 2 g of PP with 5 Fe spheres at 30 Hz at RT and 80, 120, or 160 °C. (C) C_1–10_ hydrocarbon yield obtained after 1 h of milling 2 g of PP with 5 Fe spheres at 30 Hz at RT and 80, 120, or 160 °C.

In the non-catalytic case, small amounts of C_5–10_ hydrocarbons were detected upon starting the heating (Fig. 4A, Fig. S8). However, we only observed them initially instead of continuously and therefore consider them as residual species from polymer production or processing, rather than direct products of thermal pyrolysis at these low temperatures ≤160 °C. Upon starting the ball milling, we observed the formation of products in a more continuous manner, and also lighter species, such as propene, as a product of backbone scission and subsequent depolymerization (Fig. 4B). However, propene selectivity decreases with higher temperature. Instead, higher pyrolysis products and more methane are observed (Fig. 4C). A detailed discussion of the effect of temperature on polymer backbone cleavage and the formation rates of C_1–3_ hydrocarbons can be found elsewhere.^19^ To summarize, temperature-induced softening of PP^52^ leads to less effective translation of macroscopic forces to backbone bonds. This causes lower rates of mechano-chemical chain cleavage, lower concentrations of mechano-radical intermediates, and therefore a decrease in product formation rates when increasing the temperature to 80 and 120 °C. In contrast, increasing the temperature even further to 160 °C causes higher production rates again, presumably due to a combination of thermo- and mechano-chemical activation. High temperatures are accompanied by a shift in selectivity, with methane formed as a product of over-cracking and higher pyrolysis products which are related to radical transfer and scission reactions after initial backbone bond cleavage.^19,26^

**Fig. 5:**
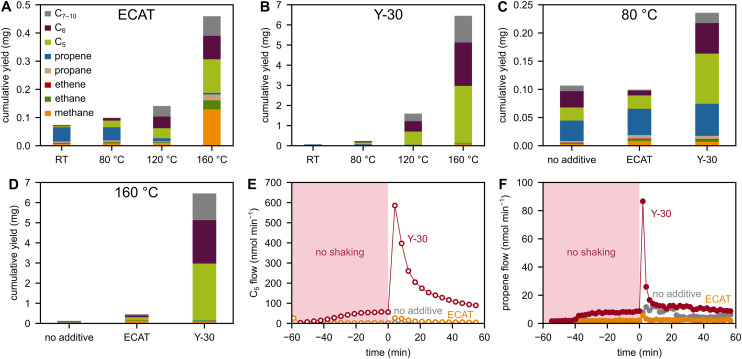
Catalytic high-temperature ball milling experiments. C_1–10_ hydrocarbon yield obtained after 1 h of milling 2 g of PP with 5 Fe spheres at 30 Hz at RT and 80, 120, or 160 °C with (A) 100 mg of ECAT and (B) 100 mg of Y-30. C_1–10_ hydrocarbon yield obtained after 1 h of milling 2 g of PP with 5 Fe spheres at 30 Hz at (C) 80 and (D) 160 °C with and without 100 mg of ECAT or Y-30. See Fig. S9 for milling at 120 °C. (E) C_5_ flow during milling of 2 g of PP with 5 Fe spheres at 30 Hz at 120 °C with and without 100 mg of ECAT or Y-30. (F) Propene flow during milling of 2 g of PP with 5 Fe spheres at 30 Hz at 160 °C with and without 100 mg of ECAT or Y-30.

Adding zeolite-based powder catalysts during ball milling of PP at elevated temperatures influences both the total rates and selectivities of hydrocarbon production (Fig. 5A–D). Similar to non-catalytic experiments, propene selectivity is inversely related to temperature, which is plausible considering that thermo-catalytic pyrolysis of PP over FCC catalysts at 450 °C produces <10 wt% propene.^2^ However, overall hydrocarbon productivity is clearly increased during catalytic milling at high temperatures compared to heating without milling (*vide infra*). This indicates a synergy of mechano- and thermo-chemical effects, and mechano-chemical activation seems necessary to induce high catalyst activity at the given temperatures.

**Fig. 6:**
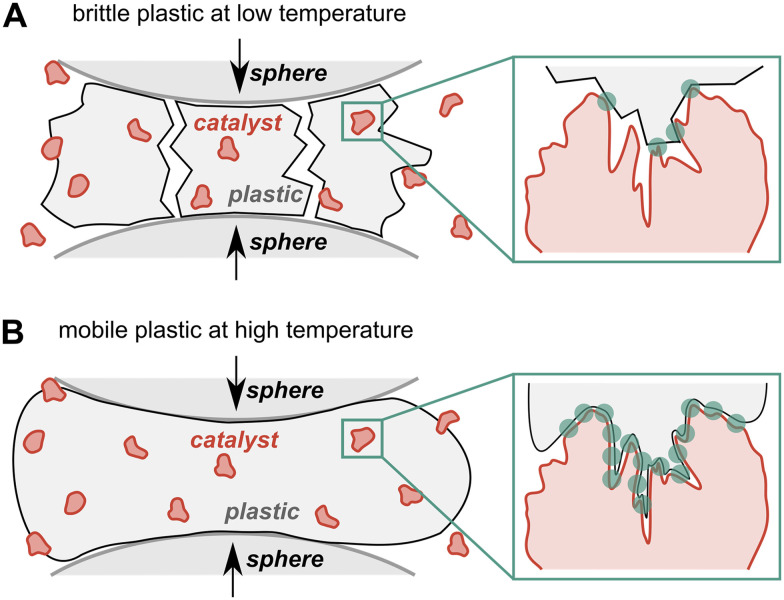
Illustration of different mechano-chemical activation modes when milling (A) solid and (B) molten PP with a catalyst powder. Contact points of the catalyst and plastic are indicated with dots.

Activity when milling with both Y-30 and the ECAT is especially pronounced for the experiments performed at 160 °C. In addition to an inherent increase in catalytic cracking activity at more elevated temperatures, we believe that this boost in activity is also caused by the melting of PP (melting temperature *T*_m_ = 160 °C, see Fig. S10 for photographs). This could cause increased infiltration of the catalyst system and allow the polymer to reach a higher number of active sites, thereby promoting the observed rate of cracking reactions. In contrast, while the brittle fracture of PP at temperatures below *T*_m_ can cause contacts with catalytically active sites, these contact points are much more limited in number due to the hard and relatively immobile nature of the material (Fig. 6).

We propose that ball milling can force catalyst–polymer contacts by essentially squeezing the molten plastic into catalyst pores, which increases activity through more contact. Another mode of activation could be the mechano-chemical pre-scission of polymer backbones prior to catalytic conversion. This would cause a decline in molecular weight and an increase in mobility of the plastic melt to infiltrate the catalyst system deeper, thereby overcoming transport limitations.^4,6^

When comparing the temperature-dependent reactivity of Y-30 and the ECAT, several key differences are observed in the most important descriptors of catalyst performance, namely activity, stability, and selectivity.

Regarding activity: Y-30 is much more active than the ECAT, both initially and during sustained milling, forming significantly higher cumulative C_1–10_ hydrocarbon yields at 80, 120, and 160 °C (Fig. 5C and D). Even during pre-heating without milling, significant product generation is observed from Y-30 already at 120 °C, which is not the case when using the ECAT or no catalyst (Fig. 5E). The higher activity of Y-30 is most likely rooted in the high intrinsic thermo-catalytic cracking reactivity of zeolite Y which can be utilized efficiently when PP is mobile enough to reach active sites. The activity of the ECAT is lower, because zeolite Y is only present in the ECAT in a diluted and partially deactivated form.

Regarding selectivity: Y-30 produces large amounts of C_5–6_ hydrocarbons which are typical pyrolysis products (Fig. 5B). In contrast, the ECAT directs selectivity towards methane which is connected to the lower overall productivity (Fig. 5A).

Regarding stability: despite being generally more active at higher temperatures, both Y-30 and the ECAT do not show sustained hydrocarbon formation rates when milling at 120 and 160 °C (Fig. 5E). This is especially evident for high-temperature experiments with Y-30. For example, when starting the milling with Y-30 at 120 or 160 °C, production of propene spikes to extremely high levels, but decreases to non-shaking conditions after *ca.* 20 min (Fig. 5F). The production of heavier hydrocarbons seems to proceed somewhat longer at catalytic levels, but this is likely caused by their longer residence time in the reactor setup due to their lower volatility. We hypothesize two main reasons for the observed deactivation.

(i) We believe that the destruction of zeolite structures (*vide supra*) by mechanical forces and the resulting loss of activity is exacerbated at higher temperatures due to the less effective cushioning of the plastic material. When milling at room temperature, the relatively ductile but resilient plastic material can protect the harder zeolite against impacts by absorbing the majority of shock load. The plastic effectively acts like a cushion for the zeolite material. At high temperatures, the plastic becomes too ductile, or even molten, so that its structural integrity is not enough to withstand mechanical impacts as effectively. Therefore, it becomes too soft to be an effective cushion, resulting in zeolite being crushed more than at lower temperatures leading to faster deactivation.

(ii) While squeezing polymers into catalyst pores increases initial contact and activity, we believe that this very effect could block the active sites of the zeolite material. At the applied temperatures, the polymer is relatively non-mobile and blocks products from leaving the pores. This can result in unwanted follow-up reactions that could lead to the deposition of carbonaceous species, causing further pore blockage. On the one hand, the plastic is soft enough to be squeezed into pores by mechanical forces, especially considering potential local frictional heating under impact leading to temporarily increased mobility. On the other hand, the plastic is afterwards stuck in the pores and creates a barrier for exiting products and the further incoming plastic material. If operating below the melting point, PP could also crystallize in the pores, exacerbating the effect.

To summarize, we observed a clear synergy between mechano- and thermo-catalysis when ball milling PP with zeolite-based catalysts. However, small-pore zeolites do not seem to be ideal materials for mechano-chemical high-temperature conversion due to their limited mechanical stability under harsh direct impacts by ball milling and the propensity for pore blockage.

### 3.3 Catalytic kneading of polypropylene at elevated temperatures

While ball milling can offer high mechanical force inputs to unlock synergies between thermo- and mechano-catalysis, it also leads to catalyst deactivation due to relatively aggressive impacts. To investigate a different mechano-chemical geometry which does not subject the materials to harsh instantaneous impacts but distributes energy input into the reactor system more uniformly, we performed experiments on a kneading instrument. In addition, utilization of this system allowed the use of higher plastic loadings than a ball mill, making it interesting for larger-scale operations. Furthermore, insights generated on a kneader geometry can be used to derive design principles such as residence times, temperatures, and catalyst loading for continuous mechano-catalytic extrusion processes. The reason for the conceptual similarity of kneading and extrusion is the method of force input with counterrotating elements exposing the molten plastic to high shear forces. However, unlike extrusion, kneading in our case was not performed in a continuous mode with the polymer transported through the system, but in semi-batch mode.

For kneading experiments, we used 46 g of PP (*M*_n_ = 37 000 g mol^−1^, *M*_w_ = 246 000 g mol^−1^) together with 2.3 g of ECAT in a Haake Rheomix PolyLab system (net volume of the mixing chamber: 69 ml) equipped with counterrotating mixing elements. The temperature was measured using a thermocouple reaching into the plastic melt, and the torque needed for maintaining a set kneading rate (rpm) was continuously recorded. The milling chamber was modified with holes and gas connections to allow for a continuous gas flow. Gaseous products were eluted from the kneading chamber using a N_2_ flow of 250 ml min^−1^ and analyzed with a portable mass spectrometer (MS). The MS was used to detect hydrocarbon fragments, such as *m*/*z* = 27 and *m*/*z* = 41 reflecting the ethenyl (C_2_H_3_^+^) and propenyl (C_3_H_5_^+^) fragments, respectively. These fragments can stem from a plethora of different gaseous hydrocarbon products generated during degradation of PP. We use their intensity to determine overall catalytic efficiency. Condensable oils were collected using a glass condenser in an ice bath and analyzed *via* offline gas chromatography (GC). The residual polymer material was collected and analyzed *via* gel permeation chromatography (GPC).

**Fig. 7:**
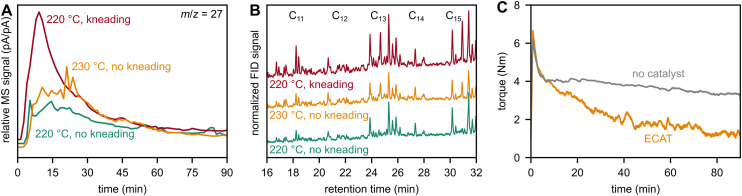
Kneading experiments performed on 46 g of PP with or without 2.3 g of ECAT. (A) Relative MS signals (normalized with respect to the nitrogen signal at *m*/*z* = 28) of *m*/*z* = 27 (C_2_H_3_^+^ fragment) recorded during kneading with the ECAT (220 °C at 50 rpm, 230 °C at 5 rpm, 220 °C at 5 rpm). (B) Gas chromatograms recorded with a flame ionization detector (FID) of condensed oils obtained after kneading for 90 min with the ECAT (220 °C at 50 rpm, 230 °C at 5 rpm, 220 °C at 5 rpm). The gas chromatograms shown are normalized with respect to butyl decanoate as an internal standard (not shown in the graph). (C) Torque required for a kneading rate of 50 rpm when kneading at 220 °C with and without the ECAT, see Fig. S14 for torques at 5 rpm.

Initial reactions were performed at a set temperature of 220 °C with a kneading rate of 5 or 50 rpm. 5 rpm was chosen to represent the non-mechano-chemical case instead of 0 rpm to minimize the mechanical energy intake while ensuring an even temperature distribution in the reactor by gentle mixing. Without a catalyst, no products were detected at 220 °C (Fig. S11). With a catalyst at 5 rpm, gaseous hydrocarbon products were formed already at this temperature and fragments with, *e.g.*, *m*/*z* = 27 (Fig. 7A) and 41 (Fig. S12), were detected. When kneading at 50 rpm, the mechanical energy intake is much higher due to the vigorous viscous kneading, and higher product signals were detected, indicating a beneficial effect of mechanical forces. However, due to the vigorous kneading and the high energy intake, the temperature during kneading at 50 rpm increased from 220 to 230 °C, rendering the comparison to experiments at 5 rpm difficult, where no such temperature increase was observed. To decouple mechanical and thermal contributions, another experiment was performed at 230 °C and 5 rpm. The product signals were lower than those in the previous experiment at 50 rpm, clearly showing a beneficial effect of mechanical forces and a synergy between thermo- and mechano-catalysis (Fig. 7A and S12).

Besides gas phase products, we condensed oils in an ice-cooled condenser and analyzed them *via* GC with flame ionization detection (Fig. 7B). Catalytic kneading at 50 rpm at a set temperature of 220 °C produces higher yields of condensable hydrocarbon species than catalytic kneading at 5 rpm and 220 or 230 °C, as can be seen *via* the higher intensities of hydrocarbon species in the GC. This is in line with the beneficial effect of kneading on gaseous hydrocarbon production and again illustrates a synergy between mechano- and thermo-catalysis. Due to the relatively low reaction temperatures, however, most of the polymer remains in the reactor after kneading. We performed GPC to observe changes in the molar mass distribution of these residues due to mechano-catalytic activation. In accordance with MS and GC data, kneading as opposed to not kneading causes additional degradation of polymer chains to lower molar mass fragments (Fig. S13A, Table S3).

In addition to an increase in small hydrocarbon productivity, addition of a catalyst also changes the rheological behavior of the remaining plastic material. More specifically, the presence of the ECAT influences the torque during kneading which is needed to maintain the set kneading rate of 50 rpm at a set point of 220 °C (Fig. 7C). In both cases, the temperature increased to 230 °C due to the mechanical energy intake, as described above, and the initial torque was virtually identical. In both cases, the torque decreases over time, but this decline is much more drastic when kneading with the ECAT compared to without. The decline in the non-catalytic case is attributed to a lowering in viscosity of the material due to chain scission events caused by thermo-mechanical degradation. When using a catalyst, however, the acidic catalyst initiates additional chain cleavage events, leading to a much faster decrease in viscosity, torque, and therefore kneading efficiency. This is also reflected in the molar mass distributions (Fig. S13B, Table S3) of the polymer residue. On the one hand, high levels of catalytic activity are desired for fast and efficient degradation of the polymer material to small hydrocarbon products. On the other hand, the decrease in torque decreases the energy input. Therefore, it is difficult to drive mechano-chemical reactions at later stages of the reaction. This aspect is also important to consider when choosing residence times in future catalytic extrusion processes.

## 4. Conclusions

Catalytic pyrolysis and mechano-chemical depolymerization are two evolving chemical recycling techniques for polymers. While zeolitic pyrolysis catalysts are highly active but lack selectivity, mechano-chemistry is more selective but suffers from low yields. We therefore investigated the combination of the two technologies to evaluate potential synergies. Zeolites were found to be active catalysts during ball milling of PP, and their activity at ambient temperature scales with acid site density. While this qualifies zeolite materials as mechano-catalysts to increase yields during mechano-chemical recycling, their activity at high ball mill loadings is not high enough to reach appreciable conversion levels. Further investigation into reaching more quantitative polymer conversion is therefore necessary. In addition, zeolites deactivate quickly due to the forceful mechanical impacts leading to framework collapse. This was also the case at higher temperatures up to 160 °C, where the thermo-catalytic cracking activity of zeolite materials is enhanced but the catalyst is less effectively protected from impacts due to the molten state of the polymer material. The collapse of the zeolite structure under harsh impacts is a fundamental problem and is difficult to overcome during mechano-chemical conversion in the ball mill, where extreme forces are necessary to activate the very stable carbon–carbon backbone bonds of the plastic.

As a strategy to circumvent deactivation, we employed direct mechano-catalysis where grinding spheres possess catalytically active surface sites. More specifically, we attached a zeolite material to the surface of pre-roughened grinding spheres. This protects the framework from forceful collisions with other grinding tools, while allowing contact with polypropylene, leading to more sustained mechano-catalytic activity while using significantly lower amounts of zeolite.

Catalytic kneading in the molten polymer state could also avoid the destruction of the zeolite structure, because the material is not impacted by frontal impacts, but energy is dissipated more uniformly over space and time. In addition, kneading offers an alternative mode of mechano-chemical activation, relying on applied shear stress rather than direct compression of the material. Kneading is also easily combined with thermal energy input. We observed enhanced depolymerization activity with kneading, illustrating the benefit of combined thermo- and mechano-chemistry. However, effects were most strongly pronounced in the initial phases of kneading, while force intake during later stages of kneading was limited by the loss of polymer chain length and viscosity.

To conclude, the combination of zeolite-based catalyst powder with mechano-chemical activation indeed caused synergistic effects. However, there are fundamental problems connected to zeolite stability. In this study, we show that direct functionalization of grinding tools or catalytic kneading can possibly overcome this issue. However, additional challenges in maintaining a high mechanical force input must be addressed before zeolite-based mechano-catalytic conversion becomes a viable chemical recycling strategy.

## Author contributions

Conceptualization: IV; methodology: IV, AHH, NK, FP, TW; investigation: AHH, CLS, HP, LS, VMOG; funding acquisition: IV, NK; project administration: IV; supervision: IV; writing – original draft: AHH; writing – review & editing: AHH, CLS, HP, LS, VMOG, NK, FP, TW, IV.

## Conflicts of interest

IV and AHH are employees of Utrecht University and inventors on two patent applications by Universiteit Utrecht Holding B.V. entitled “Mechanochemical catalytic depolymerisation”, EP22201902.8, and “Catalytically functionalized grinding media for mechanochemical depolymerization”, EP24170863.5.

## Supplementary Material

CY-015-D5CY00935A-s001

## Data Availability

Data for this article, including gas chromatography, mass spectrometry, gel permeation chromatography, thermogravimetric analysis, inductively coupled plasma optical emission spectroscopy, N_2_ physisorption, scanning electron microscopy, temperature-programmed desorption of NH_3_, and X-ray diffraction as well as analysis codes are available *via* the Yoda repository at https://doi.org/10.24416/UU01-21H8W5. Supplementary information (SI): containing Fig. S1–S14 and Table S1–S3. See DOI: https://doi.org/10.1039/d5cy00935a.
