# Supplementary material for: Mechano-catalytic conversion of polypropylene over zeolite-based materials
Source: Catal Sci Technol. 2025 Oct 24;15(24):7525–38. doi: 10.1039/d5cy00935a (PMC12598732; doi:10.1039/d5cy00935a)
Supplement: CY-015-D5CY00935A-s001 [file CY-015-D5CY00935A-s001.pdf]

Supplementary Information (SI) for Catalysis Science & Technology.
This journal is © The Royal Society of Chemistry 2025

Supporting Information

Mechano-catalytic conversion of polypropylene over zeolite-based materials

Adrian H. Hergesell[1], Claire L. Seitzinger[1], Hubert Pasternak[1], Laura Seidling[1], Viviana M. Ospina Guarin[1], Nicole Karpensky[2], Florian Puch[3,4], Thomas Welzel[3], Ina Vollmer[1]*

[1]*Inorganic Chemistry and Catalysis Group, Institute for Sustainable and Circular Chemistry, Utrecht University, The Netherlands*

[2]*carboliq GmbH, Remscheid, Germany*

[3]*Thüringisches Institut für Textil- und Kunststoff-Forschung e.V., Rudolstadt, Germany*

[4]*Plastics Technology Group, Faculty of Mechanical Engineering, Technische Universität Ilmenau, Germany*

E-mail: i.vollmer@uu.nl

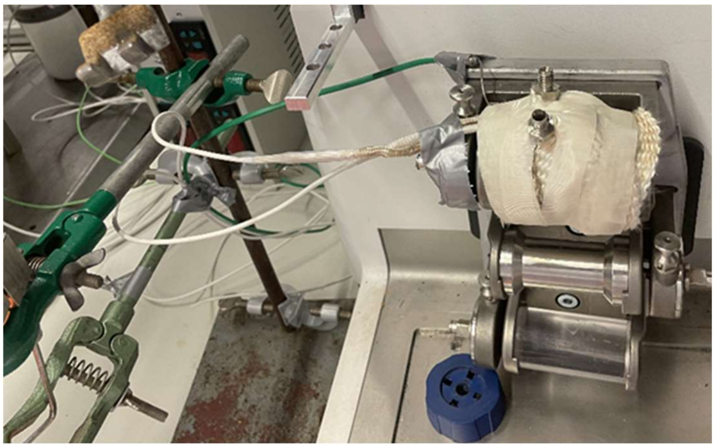

Fig. S1. Setup used for high-temperature ball milling experiments. The container was wrapped in heating wire, a thermocouple was attached, and glass fiber insulation tape was secured with heat-resistant tape.

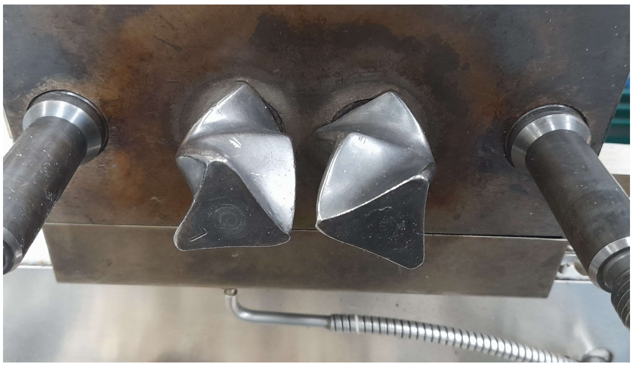

Fig. S2. Photograph of kneading screws.

Table S1. $C_{1–10}$ hydrocarbon yield obtained after 1 h of milling 1 g of PP with 6 Fe spheres at 30 Hz with 7 wt% of Y-30: Three replicate experiments, average, and standard deviation.

experiment	yield (mg g^{-1})							
	methane	ethane	ethene	propane	propene	C_5	C_6	$C_{7–10}$
1	0.0103	0.0035	0.0010	0.0049	0.0499	0.0155	0.0033	0.0017
2	0.0081	0.0033	0.0008	0.0046	0.0548	0.0229	0.0017	0.0016
3	0.0121	0.0044	0.0009	0.0057	0.0675	0.0200	0.0014	0.0016
average	0.0101	0.0037	0.0009	0.0051	0.0574	0.0195	0.0021	0.0016
std. deviation	0.0020	0.0006	0.0001	0.0006	0.0091	0.0037	0.0010	0.0001

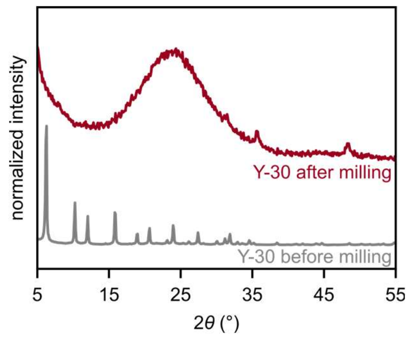

Fig. S3. X-ray diffractograms of zeolite Y before and after milling of 0.5 g of Y-30 for 10 min at 30 Hz with 6 Fe spheres.

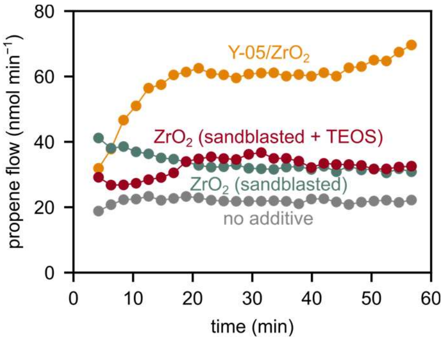

Fig. S4. Propene flow during milling of PP with 6 ZrO_2-based spheres at 30 Hz: untreated ZrO_2, sandblasted ZrO_2, sandblasted and TEOS-treated ZrO_2 (without zeolite), and Y-05/ZrO_2 grinding spheres.

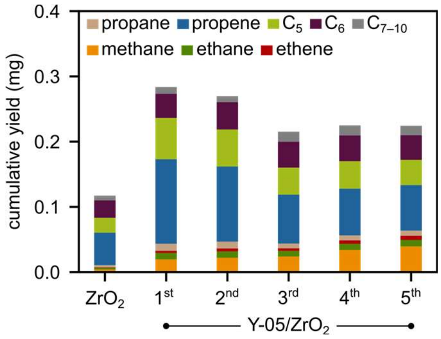

Fig. S5. Catalyst recycling experiments. $C_{1\text{–}10}$ hydrocarbon yield obtained after 1 h of milling of PP with six untreated ZrO_2 or consecutively used Y-05/ZrO_2 grinding spheres at 30 Hz. In between runs, catalytic Y-05/ZrO_2 spheres were removed from the residual plastic material, washed with deionized water, and dried in air.

Table S2. Catalyst recycling experiments. Relative propene and C_{1-10} hydrocarbon yield (normalized with respect to yields during 1st catalytic cycle) obtained after 1 h of milling of PP with six untreated ZrO_2 or consecutively used Y-05/ZrO_2 grinding spheres at 30 Hz. In between runs, catalytic Y-05/ZrO_2 spheres were removed from the residual plastic material, washed with deionized water, and dried in air.

usage cycle	relative yield (%)	
	propene	C_{1-10}
1st	100	100
2nd	89	95
3rd	58	76
4th	55	79
5th	54	79
non-catalytic	38	41

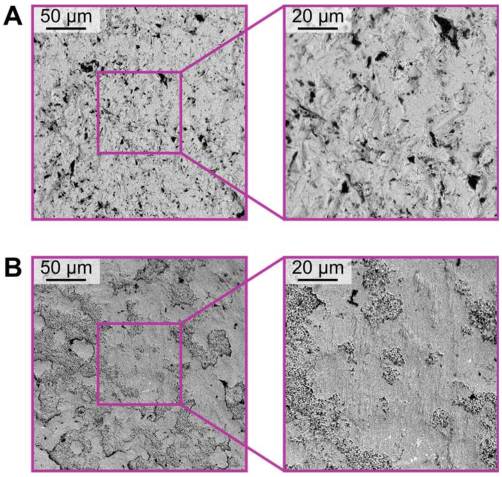

Fig. S6. Scanning electron micrographs of **(A)** a fresh 10 mm Y-05/ZrO_2 grinding sphere and **(B)** a 10 mm Y-05/ZrO_2 grinding sphere after two milling experiments with 6 grinding spheres and 1 g PP at 30 Hz.

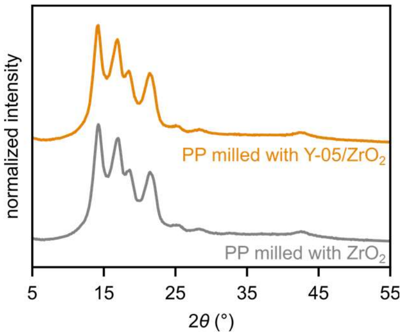

Fig. S7. X-ray diffractograms of PP after 1 h of milling with 6 ZrO_2 spheres and 6 Y-05/ZrO_2 spheres at 30 Hz.

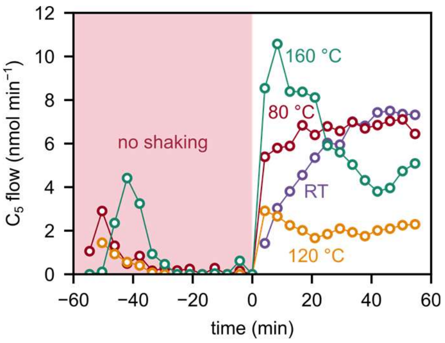

Fig. S8. C_5 flow during milling of 2 g of PP with 5 Fe spheres at 30 Hz at RT and 80, 120, or 160 °C.

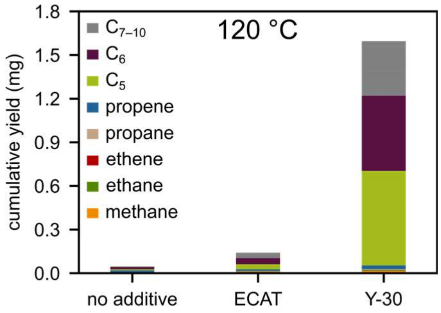

Fig. S9. $C_{1–10}$ hydrocarbon yield obtained after 1 h of milling 2 g of PP with 5 Fe spheres at 30 Hz at 120 °C with and without 100 mg of ECAT or Y-30.

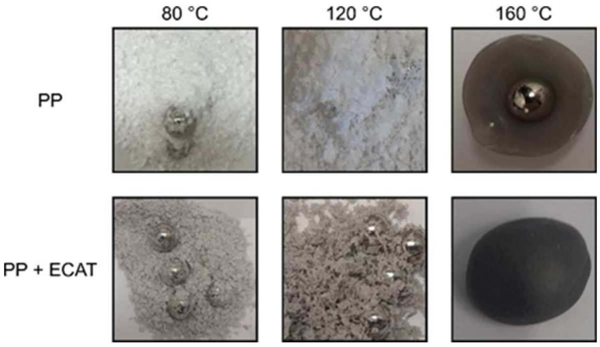

Fig. S10. Photographs of milling residue after 1 h of milling 2 g of PP with 5 Fe spheres at 30 Hz at 80, 120, or 160 °C with and without 100 mg of ECAT. While milling at 80 and 120 °C preserves the powder morphology, milling at 160 °C leads to a melting of the plastic both with and without catalyst.

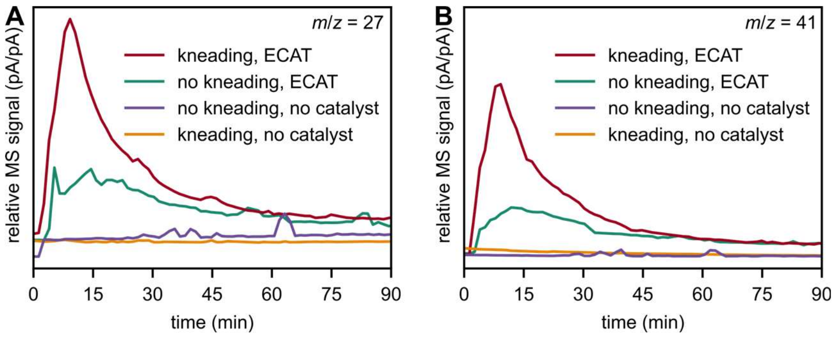

Fig. S11. Relative MS signals (normalized with respect to the nitrogen signal at m/z = 28) recorded during kneading of 46 g of PP with and without 2.3 g of ECAT at 220 °C at 5 or 50 rpm: **(A)** m/z = 27 ($C_2H_3^+$ fragment) and **(B)** m/z = 41 ($C_3H_5^+$ fragment).

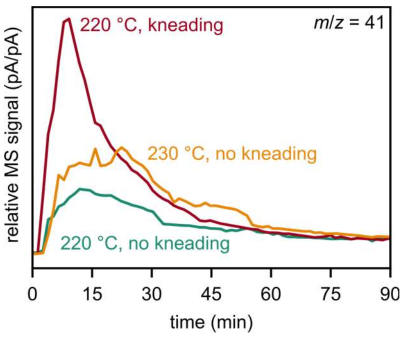

Fig. S12. Relative MS signals of m/z = 41 ($C_3H_5^+$ fragment, normalized with respect to the nitrogen signal at m/z = 28) recorded during kneading of 46 g of PP with 2.3 g of ECAT at 220 or 230 °C at 5 or 50 rpm.

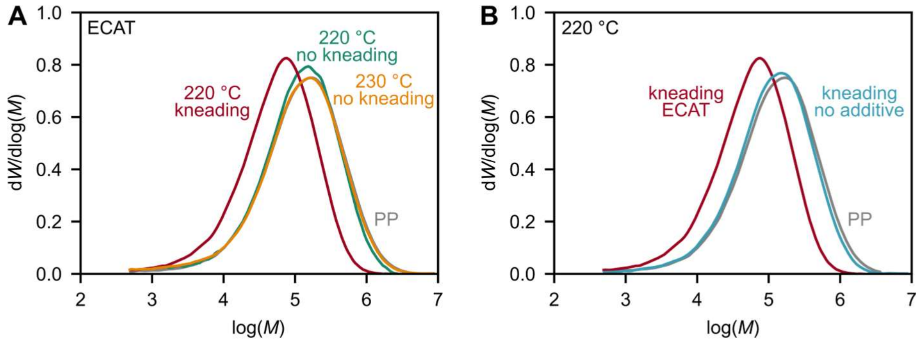

Fig. S13. (A) Molar mass distributions of pristine PP, and after kneading of 46 g of PP with 2.3 g of ECAT (220 °C at 50 rpm, 230 °C at 5 rpm, 220 °C at 5 rpm). **(B)** Molar mass distributions of pristine PP, and after kneading of 46 g of PP with and without 2.3 g of ECAT at 220 °C and 50 rpm.

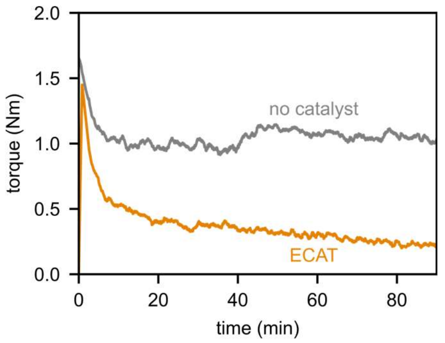

Fig. S14. Torque required for a kneading rate of 5 rpm when kneading at 220 °C with and without ECAT.

Table S3. Number average molar mass (M_n), weight average molar mass (M_w), and dispersity (M_n/M_w) of pristine PP, and after kneading of 46 g of PP with or without 2.3 g of ECAT (220 °C at 50 rpm, 230 °C at 5 rpm, 220 °C at 5 rpm).

	M_n (g mol^{-1})	M_w (g mol^{-1})	M_w/M_n (–)
pristine	37,000	246,000	6.64
220 °C, no kneading, ECAT	44,000	210,000	4.75
220 °C, kneading, ECAT	20,000	99,000	4.85
230 °C, no kneading, ECAT	30,000	240,000	8.10
220 °C, kneading, no additive	37,000	217,000	5.80
